# Patient-derived prostate organoids identify MAOA as a disease severity-associated molecular marker in chronic pelvic pain syndrome

**DOI:** 10.1038/s41598-026-53351-4

**Published:** 2026-05-19

**Authors:** Hiroyuki Kitano, Yohei Sekino, Kazuma Yukihiro, Mai Okazaki, Naofumi Nomura, Tomoya Hatayama, Hiroyuki Shikuma, Yoshinori Nakano, Shinsaku Tasaka, Kyosuke Iwane, Ryo Tasaka, Yuki Kohada, Syunsuke Miyamoto, Miki Naito, Kohei Kobatake, Nagisa Morihara, Nobuyuki Hinata

**Affiliations:** 1 Department of Urology, Graduate School of Biomedical and Health Sciences, Hiroshima University, Hiroshima, 734-8551 Japan; 2 Department of Clinical Practice and Support, Hiroshima University Hospital, Hiroshima, 734-8551 Japan

**Keywords:** Chronic pelvic pain syndrome, Prostate organoids, Monoamine oxidase A, CALB1, Biomarkers, Biomarkers, Diseases, Medical research, Urology

## Abstract

Chronic pelvic pain syndrome (CPPS) is a multifactorial condition with unclear pathophysiology and a lack of objective biomarkers for assessing disease activity. To investigate its molecular basis, we generated patient-derived prostate organoids from biopsy tissues of nine patients with CPPS, categorized as mild, moderate, or severe according to the NIH CP Symptom Index scores. The organoids were treated with Eviprostat or tadalafil, followed by transcriptomic profiling, quantitative RT-PCR validation, and immunohistochemical analysis of candidate genes. Among the differentially expressed genes, *monoamine oxidase A* (MAOA) and *calbindin-D28K* (CALB1) were consistently associateds with symptom severity. Expression of both genes decreased in organoids and prostate tissues as symptom severity increased, whereas serum MAOA levels were significantly elevated in patients with severe CPPS. These findings suggest that MAOA and CALB1 reflect molecular alterations linked to symptom intensity and may serve as potential biomarkers for CPPS. Furthermore, patient-derived prostate organoids offer a valuable experimental platform for elucidating disease mechanisms and evaluating therapeutic interventions in prostatitis-related disorders.

**Supplementary Information:**

The online version contains supplementary material available at 10.1038/s41598-026-53351-4.

## Introduction

Chronic pelvic pain syndrome (CPPS) often presents with urinary symptoms such as frequency, urgency, and pelvic, perineal, and genital pain, significantly impairing the patients’ quality of life^1,2^. Chronic prostatitis (CP)/CPPS, National Institutes of Health (NIH) category III, is a common genitourinary diagnosis in men and is defined as the “presence of genitourinary pain in the absence of uropathogenic bacteria, as detected by standard microbiological methodology”^3^.

However, its etiology remains unclear, and its pathophysiology cannot be explained by a single mechanism. Potential causes include infection, lower urinary tract dysfunction, and prostate inflammation^4^.

The Multidisciplinary Approach to the Study of Chronic Pelvic Pain (MAPP) Research Network initiated a series of studiesaimed at exploring the causes and potential treatments of CPPS from multiple perspectives, with the goal of characterizing CPPS as a systemic condition that potentially involve multiple etiologies^5^. Molecular and pathophysiological factors have been studied, resulting in a comprehensive research strategy integrating these multidisciplinary findings.

Organoids are innovative three-dimensional in vitro culture systems that form self-renewing, near-physiological tissues driven by stem cells using defined niche factors^6^.

Organoids are composed of multiple organ-specific cell types derived from stem cells and organ-specific progenitor cells^7^. Organoids represent a well-established in vitro model that closely mimics in vivo conditions, retaining stem cells, making them a valuable platform for analyzing cancer stem cell functions^8^. This technology has been applied to various types of cancer tissues, including gastric cancer^9,10^, and studies have demonstrated that cancer-derived organoids faithfully retain the majority of the genetic and histological characteristics of their original tumors^11^. To our knowledge, no studies have utilized organoids as in vitro models for CP/CPPS.

Therefore, this study aimed to generate organoids from prostate tissues obtained from patients with CPPS (CPPS organoids; CPPSOs) and to identify the molecules potentially involved in the pathogenesis or disease activity of CPPS.

## Results

### Establishment of CPPSOs

**Fig. 1:**
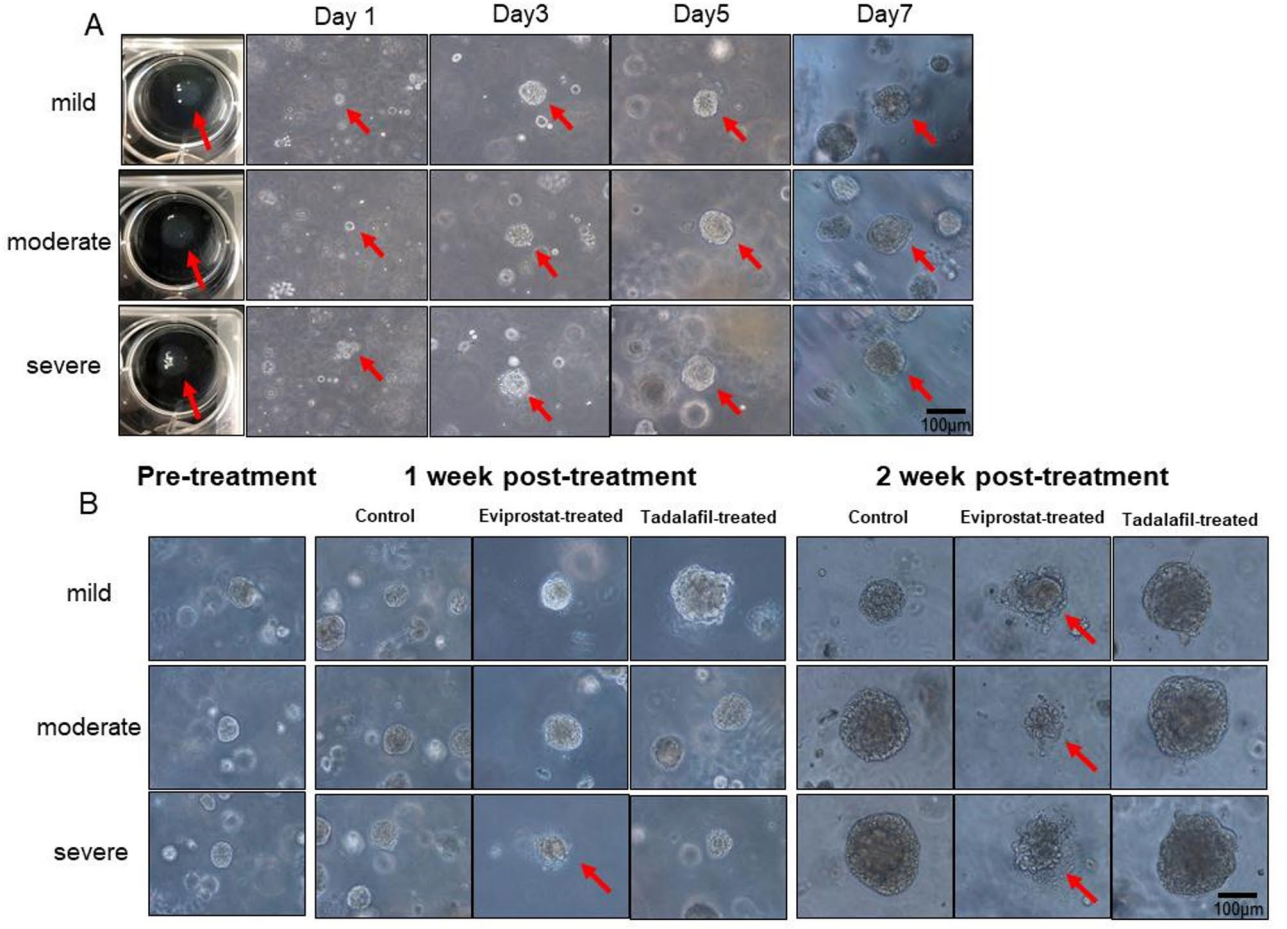
Establishment and morphology of CPPS organoids (CPPSOs). (**A**) CPPS organoids were generated from nine patients classified as mild (*n* = 4), moderate (*n* = 2), or severe (*n* = 3) based on NIH-CPSI scores. No marked morphological differences were observed among CPPSOs across severity categories. (**B**) Organoids were cultured for 1 week and then treated with vehicle (control), Eviprostat (50 µg/mL), or tadalafil (10 µM). Eviprostat-treated organoids exhibited irregular and disrupted morphology compared with Tadalafil-treated organoids and controls.

Nine patients diagnosed with CPPS were categorized according to their NIH CP Symptom Index (NIH-CPSI) scores: four with mild, two with moderate, and three with severe symptoms. CPPSOs were successfully established in all cases. No obvious morphological differences were observed among CPPSOs according to symptom severity (Fig. 1a). Organoids were cultured for one week and then divided into three groups: control, Eviprostat-treated (50 µg/mL), and tadalafil-treated (10 µM). Compared with tadalafil-treated organoids, viprostat-treated organoids exhibited characteristic morphological irregularities (Fig. 1b).

### Microarray analysis of CPPSOs

Microarray analysis was performed on CPPSOs derived from nine cases (four mild, two moderate, and three severe). Hierarchical clustering revealed that mild and moderate CPPSOs exhibited similar expression profiles (Supplementary Fig. S1). To identify key markers, we employed a multi-step filtering process based on statistical significance and biological relevance.

First, a one-way ANOVA across the experimental groups identified 468 genes with statistically significant differences (*p* ≤ 0.05). The complete list of these differentially expressed genes (DEGs) is provided in Supplementary Table S1. From this pool, we prioritized genes exhibiting a fold-change ≥ 2.0 compared to their respective controls. Subsequent Ingenuity Pathway Analysis (IPA) identified several inflammation-related pathways and associated genes, including *MAOA*, *IL-6*, *CALB1*, and *STAG2*.

To enhance the specificity of our selection, we integrated baseline severity-dependent expression with the response to pharmacological treatments (Eviprostat and tadalafil), using the drugs as “functional filters” to isolate dynamically regulated genes. Through this multi-conditional filtering, *MAOA* was consistently identified as a core inflammation-related gene across all analyses and our previous independent studies, leading to its selection as the primary candidate. While *CALB1* demonstrated clear severity-dependent expression, genes such as *STAG2* and *IL-6* were excluded from further primary investigation, as they failed to show consistent associations with both clinical severity and pharmacological response across the cohorts.

All analyses were conducted using *the Transcriptome Analysis Console* (TAC, version 4.0.1.36; Thermo Fisher Scientific, Waltham, MA, USA), *GeneSpring* (version 14.9.1 – GX – PA; Agilent Technologies, Santa Clara, CA, USA), and *Ingenuity Pathway Analysis* (IPA; Tomy Digital Biology Co., Ltd., Tokyo, Japan).

### mRNA expression in control CPPSOs

**Fig. 2:**
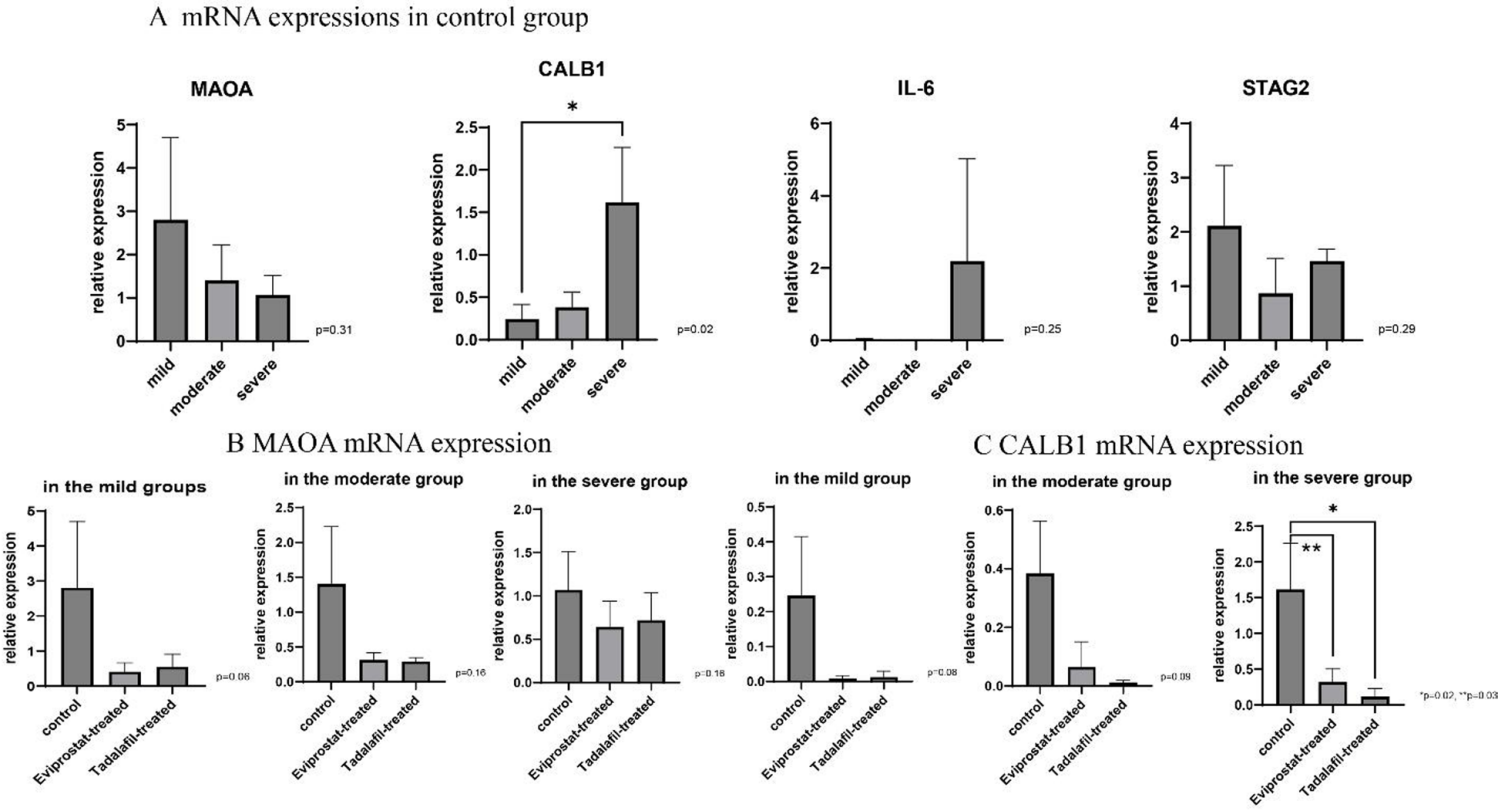
mRNA expression and treatment response in CPPSOs. (**A**) Baseline gene expression in untreated CPPSOs. CALB1 expression was elevated in severe cases, while MAOA expression decreased with symptom severity. IL-6 and STAG2 showed non-significant trends toward higher expression in severe cases. (**B**,**C**) Following Eviprostat or tadalafil treatment, MAOA expression decreased across all severity categories (**B**). CALB1 expression decreased post-treatment, particularly in severe organoids (**C**).

Quantitative RT-PCR revealed that *CALB1* expression was significantly higher in organoids derived from patients with severe symptoms than in those with mild symptoms (*p* = 0.02), exhibiting a clear severity-dependent pattern. Conversely, *MAOA* expression showed a decreasing trend with increasing symptom severity. While preliminary analysis of *IL-6* and *STAG2* showed higher expression in severe cases, these changes were not statistically significant (Fig. 2A–C).

Following pharmacological treatment, *MAOA* mRNA levels consistently decreased with both Eviprostat and tadalafil across all symptom severity groups (Fig. 2B). *CALB1* expression also decreased, with a significant reduction in organoids from severe cases (Fig. 2C). In contrast, the responses of *IL-6* and *STAG2* to pharmacological treatment were less consistent across the groups (Supplementary Fig. S2). Specifically, *IL-6* expression increased after treatment in mild and moderate cases but decreased in severe cases, while *STAG2* showed a different trend. Due to this lack of consistency across the cohorts, we prioritized *MAOA* and *CALB1* for further investigation as more robust markers of disease state and therapeutic response.

### Immunohistochemical staining

#### CPPSOs

**Fig. 3:**
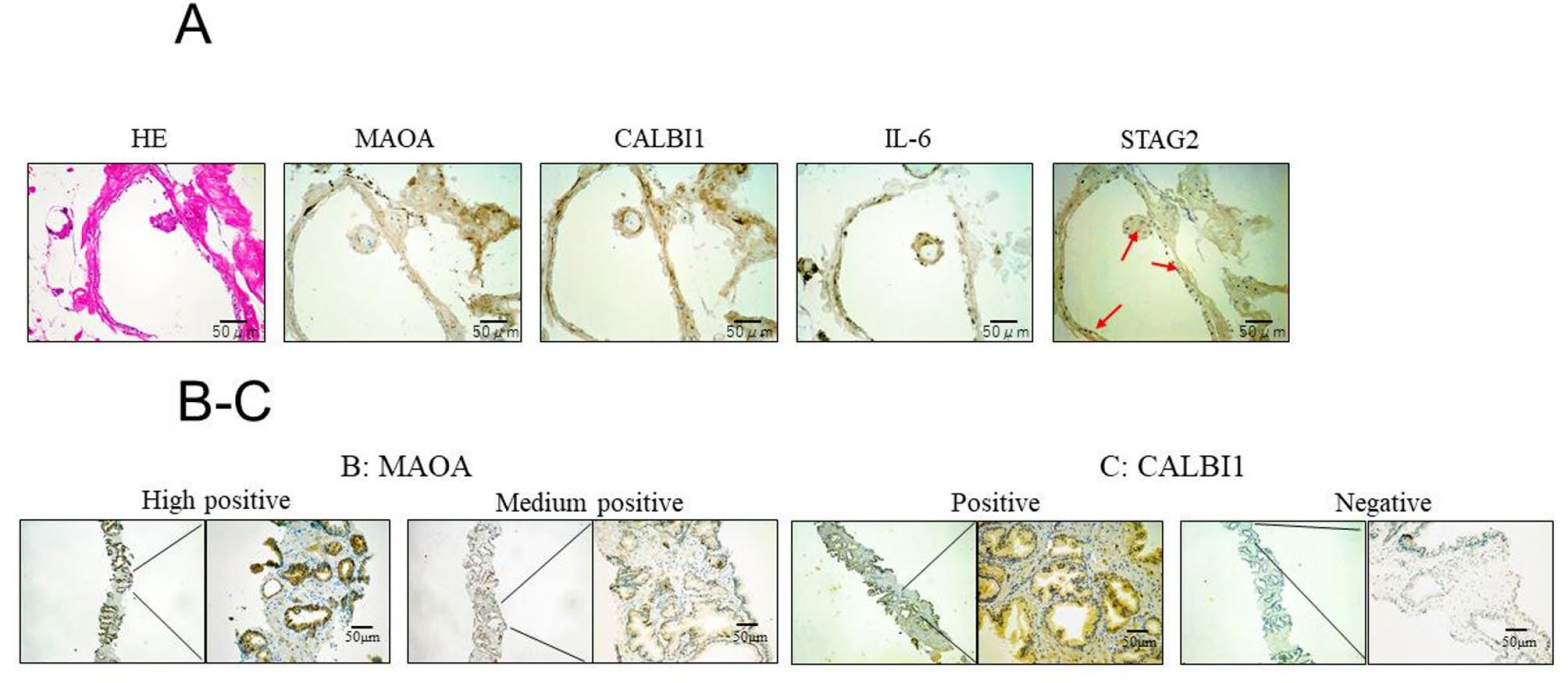
Immunohistochemical expression of candidate genes in CPPSOs and immunohistochemical expression of candidate genes in human prostate biopsy specimens. In CPPOs, MAOA and CALB1 demonstrated cytoplasmic expression in glandular epithelium. IL-6 exhibited weak cytoplasmic staining, while STAG2 showed nuclear localization (**A**). Biopsy samples from 111 CPPS patients were stratified by NIH-CPSI pain scores (≤ 0: *n* = 88; ≥1: *n* = 23). MAOA expression was lower in pain-positive cases (**B**), while CALB1 expression was more frequent in pain-positive cases (**C**).

Immunohistochemistry revealed cytoplasmic expression of MAOA and CALB1 in glandular epithelial cells, weak cytoplasmic expression of IL-6, and nuclear expression of STAG2(Fig. 3A).

#### Human prostate biopsy specimen

To validate these findings in clinical samples, we analyzed 111 patients with CPPS. Based on NIH-CPSI pain scores, samples were classified into pain-negative (score ≤ 0, *n* = 88) and pain-positive (score ≥ 1, *n* = 23) groups. MAOA expression was significantly lower, whereas CALB1 expression was significantly higher in the pain-positive group (Fig. 3B,C). In contrast, *IL-6* and *STAG2* expression showed no significant association with pain scores (Supplementary Fig S3, Supplementary Table S2). Therefore, these markers were excluded from further clinical investigation.

### MAOA activity in a human blood sample

Blood MAOA activity was measured in 13 cases that were distinct from those in which organoids were generated or in which immunohistochemistry was performed. All 13 patients were diagnosed with category IIIb CP/CPPS according to the diagnostic criteria for CP/CPPS^3^. Symptom severity was evaluated using the NIH-CPSI, with scores of 0–9 defined as mild, 10–18 as moderate, and 19–31 as severe, based on the responses to items 1–6. Among these patients, one was classified as mild, nine as moderate, and four as severe.

**Fig. 4:**
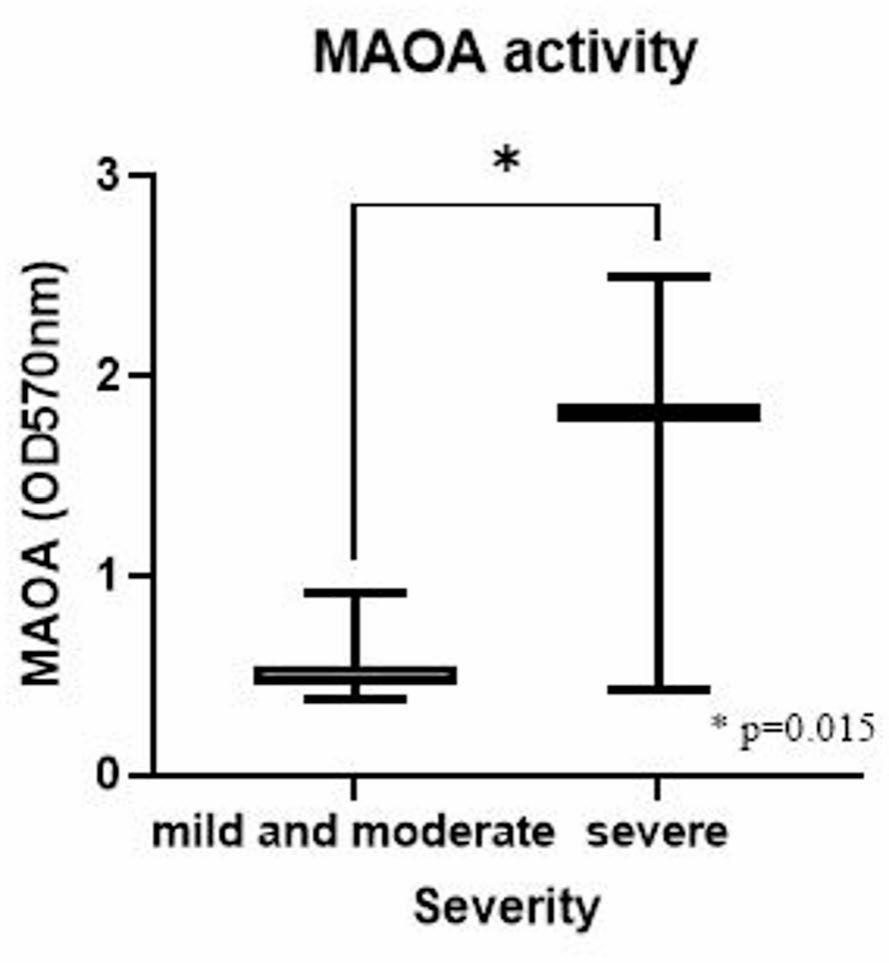
Blood MAOA activity in patients with CP/CPPS. Blood MAOA activity was assessed in 13 patients classified as mild/moderate (*n* = 10) or severe (*n* = 3). MAOA activity was significantly higher in the severe group (*p* = 0.015).

As there was only one mild case, patients with mild and moderate symptoms (*n* = 10) were combined and compared with those with severe symptoms (*n* = 3). Blood MAOA concentration was significantly higher in the severe group (*p* = 0.015; Fig. 4).

## Discussion

CPPS is a benign condition that affects approximately 8% of men in community-based studies, with incidence rates of up to 11.5% among those aged < 50 years^12,13^. In this study, CPPS prostate organoids were used to test drug effects and identify disease-related genes. Although organoid models have previously been applied in prostate cancer research^14^, to our knowledge, this is the first application of organoid technology, specifically in CPPS.

The MAPP Research Network, established by the NIH/NIDDK, aims to elucidate the pathophysiology and natural history of urologic CPPS (UCPPS), which include interstitial cystitis/bladder pain syndrome (IC/BPS) in both sexes and CP/CPPS in men^5^.

Prior analyses within the MAPP Network showed that patients with UCPPS experience more severe pain than those with other non-urological chronic overlapping pain conditions and healthy controls. They also demonstrated higher levels of anxiety, depression, perceived stress, and neuroticism, with elevated catastrophizing^15^. MAOA, which catabolizes serotonin, norepinephrine, and dopamine, has been associated with depressive disorders, and elevated circulating MAOA levels have been reported in patients with depression^16,17^. Consistent with these findings, significantly higher plasma MAOA levels were observed in patients with CPPS with more severe symptoms, suggesting its potential as a biomarker of disease severity.

In men with UCPPS, urinary MMP-9, the MMP-9/NGAL complex, and VEGF-R1 correlated positively with pain and urinary symptom severity, whereas in women, MMP-2 and these proteins showed similar associations. Although these biomarkers did not distinguish UCPPS from controls, their correlation with symptom severity suggests their utility in elucidating underlying disease mechanisms^18^.

Furthermore, IL-6 expression, previously reported to increase in rat models of prostatic inflammation, was elevated in patients with more severe symptoms, highlighting its potential as a marker of disease severity^19^. Additionally, IL-6 is reportedly associated with long-term pain in patients with CPPS, further supporting its role in disease severity^20^.

Transcriptomic analysis of the CPPSOs identified STAG2 and CALB1 as candidate genes. STAG2 is a known tumor suppressor and the causative gene in Mullegama-Klein-Martinez syndrome^21,22^, whereas CALB1 has been implicated in prostate cancer^23^. To date, neither gene has previously been associated with CPPS. In this study, MAOA consistently demonstrated a relationship with symptom severity in the organoid analysis, prostate tissue immunohistochemistry, and serum enzyme activity measurements. The ability to detect MAOA activity in blood samples suggests its potential value as a noninvasive biomarker reflecting disease activity. Eviprostat-induced morphological alterations further suggest that epithelial stress responses or inflammatory pathways may influence MAOA regulation, which is consistent with previous reports linking monoamine signaling to chronic pain modulation.

A notable finding was the inverse relationship between local MAOA mRNA expression in CPPSOs and systemic MAOA activity in serum. While MAOA was downregulated in organoids from severe cases, serum MAOA activity was significantly higher in the severe group (*p* = 0.015).

This discrepancy may be explained by the biological nature of MAOA. As a mitochondria-bound enzyme, its local reduction in organoids likely reflects mitochondrial dysfunction or a specific metabolic adaptation in the diseased prostate epithelium. On the other hand, the elevated serum activity in severe patients might represent a systemic manifestation of chronic pain and stress, or a leakage of the enzyme from damaged tissues into the peripheral circulation. Such divergent patterns between tissue-specific and systemic markers suggest that integrating local molecular signatures with serum enzyme activity could provide a more robust diagnostic framework for assessing CPPS severity.

This study has some limitations, including a small sample size and heterogeneity inherent to CPPS. In addition, organoids derived from healthy prostate tissue were not included. Therefore, the observed differences should be interpreted with caution, as they may reflect disease-associated variability rather than disease-specific mechanisms.

The consistent multilevel relevance of MAOA across assays supports its link to disease severity. Future studies incorporating larger cohorts, healthy controls, and longitudinal sampling are essential to determine whether MAOA may aid in patient stratification, treatment monitoring, and therapeutic decision-making.

In this study, CPPSOs were established using a defined culture system previously shown to preserve the phenotypic characteristics of human prostate epithelium, including luminal/basal cell markers and androgen receptor signaling. However, we acknowledge that further functional and phenotypical characterization—such as assessments of cell growth, viability, and structural organization—would be beneficial to more comprehensively validate the extent to which the disease state is manifested in vitro. While these detailed evaluations were beyond the primary scope of our biomarker discovery, the consistent expression patterns observed across our multi-cohort analysis suggest that CPPSOs effectively mirror key aspects of the clinical pathophysiology of CPPS. Importantly, patient-derived organoids provide a functional platform for evaluating pharmacological responses and offer a foundation for personalized therapeutic approaches in CPPS.

In conclusion, this study identified MAOA and CALB1 as potential molecular markers associated with symptom severity in CPPS. These findings suggest that alterations in neurotransmitter metabolism and calcium signaling pathways may contribute to disease mechanisms. Additional large-scale studies are required to validate these results and further elucidate the molecular landscape of the disease. Importantly, patient-derived CPPSOs may serve as a platform for drug screening and individualized therapy selection, thereby providing a foundation for the development of targeted treatment strategies.

## Methods

### Ethics approval

This prospective study was approved by the Institutional Review Board of Hiroshima University (E2017-0912). Written informed consent was obtained from all the patients for the establishment of organoids and sample collection from the cohort.

All experiments and analyses involving human-derived materials were performed in accordance with the relevant guidelines and regulations.

### Human tissues

Human prostate biopsy specimens were obtained from patients diagnosed with NIH category IIIb CPPS at the Department of Urology, Hiroshima University Hospital. All the patients received treatment for CP. Organoids were established from biopsy-derived tissues. Symptom severity was assessed using the NIH CP Symptom Index (NIH-CPSI), with scores of 0–9 classified as mild, 10–18 as moderate, and 19–31 as severe based on responses to items 1 through 6^24^.

Furthermore, for patients undergoing prostate biopsy, formalin-fixed, paraffin-embedded tissue blocks were prepared for immunohistochemical analysis. Based on NIH-CPSI responses to items 1 through 4 (pain domain), patients were categorized into two groups: those with CPPS-related symptoms (score ≥ 1) and those without symptoms (score = 0)^24^.

In this study, we employed a multi-cohort design to ensure reproducibility and minimize potential selection bias. Candidate genes were initially identified using a discovery cohort consisting of patient-derived prostate organoids. These findings were subsequently validated in independent patient cohorts at the tissue level using immunohistochemistry and at the systemic level using serum MAOA activity measurements.

### Establishment and culture of human chronic prostate organoids

Prostate tissues were obtained from patients categorized into three groups—mild, moderate, and severe—based on the NIH-CPSI, and organoids were subsequently established from each sample. In all cases, organoids were cultured under nine experimental conditions, comprising control, viprostat-treated, and tadalafil-treated groups, across the three symptom severity levels. Human chronic prostate organoids were established and cultured in organoid media containing niche factors as previously described^25^. Thereafter, they were observed for one week. Subsequently, they were treated with Eviprostat (50 µg/ml) and tadalafil (10 µM) and further observed for two weeks.

### Sample collection and Ribonucleic acid (RNA) preparation

Total RNA was isolated from cell pellets of organoids and frozen tissues using NucleoSpin RNA (TAKARA BIO, Shiga, Japan). Total RNA quality was assessed using the Agilent RNA 6000 Nano Kit (Agilent Technologies, Santa Clara, CA, USA) on an Agilent 2100 Bioanalyzer (Agilent Technologies, Santa Clara, CA, USA). The RNA integrity numbers of all RNA samples were greater than 7.0, which met the manufacturer’s quality criteria.

### Microarray

cRNA was synthesized from 250ng of total RNA using the GeneChip™ WT PLUS Reagent Kit (Thermo Fisher Scientific, Waltham, MA, USA), followed by the generation of biotin-labeled single-stranded DNA (ssDNA). In total, 2.3 µg of ssDNA was hybridized to the Clariom™ S Array, human (Thermo Fisher Scientific, Waltham, MA, USA) using the GeneChip™ Hybridization, Wash, and Stain Kit (Thermo Fisher Scientific, Waltham, MA, USA) in the GeneChip^®^ Hybridization Oven 645, followed by washing and staining on the GeneChip^®^ Fluidics Station 450 and scanning with the GeneChip^®^ Scanner 3000 7G, according to the manufacturer’s protocol. Quality control analysis of the Cell Intensity (CEL) files generated by scanning was performed using Transcriptome Analysis Console (TAC) software version 4.0.1.36 (Thermo Fisher Scientific, Waltham, MA, USA) with the SST-Robust Multichip Average (RMA) algorithm, which confirmed that all samples passed the hybridization control threshold and Pos vs. Neg Area under the curve (AUC) Threshold.

### Data analysis

CEL files were quantified using the Exon RMA algorithm in GeneSpring software version 14.9.1 (Agilent Technologies, Santa Clara, CA, USA), and genes with expression levels below the 20th percentile in all samples were excluded. The filtered gene list was averaged across nine experimental conditions and subjected to hierarchical clustering using Euclidean distance and Ward’s linkage method. To enhance the specificity of candidate gene selection, we employed a pharmacological perturbation strategy. By analyzing the transcriptomic shifts induced by Eviprostat and tadalafil, we sought to identify genes whose expression was not only severity-dependent but also dynamically regulated by pharmacological agents, thereby prioritizing genes with high biological relevance to CPPS pathophysiology. Statistical and fold-change (FC) analyses were performed across six pairwise comparisons (tadalafil versus control, Eviprostat versus control) groups, across three disease severity levels. One-way analysis of variance (ANOVA) with asymptotic p-value computation was applied, followed by post-hoc testing using Tukey’s HSD and multiple testing correction via the Benjamini-Hochberg method, selecting genes with adjusted p-values < 0.05 and FC > 2.

### DNA extraction and Quantitative reverse transcription-polymerase chain reaction analysis

To validate the microarray results, some genes (monoamine oxidase A [MAOA], Calbindin 1 [CALB1], interleukin-6 [IL-6], and stromal antigen 2 [STAG2]) were selected from the filtered gene list identified by data analysis, and quantitative polymerase chain reaction (qPCR) was performed using the control samples. In total, 1 µg RNA was used to synthesize cDNA by the PrimeScript first-strand cDNA Synthesis Kit (Takara Bio, Shiga, Japan). The primers used are listed in Supplementary Table S3 .

The relative expression levels were calculated using the ΔCt method, with GAPDH used as the internal control gene, and one-way ANOVA was performed to assess differences among the three severity levels.

### Reagents

Primary antibodies used were as follows: monoclonal mouse anti-MAOA (Invitrogen; Life Technologies, Carlsbad, CA), polyclonal rabbit anti-STAG2 (Invitrogen; Life Technologies, Carlsbad, CA), monoclonal mouse anti-calbindin D28K (CALB1) (Invitrogen; Life Technologies, Carlsbad, CA), monoclonal mouse anti-IL-6 (Abcam, Cambridge, UK).

### Immunohistochemical staining

Cell blocks of human chronic prostate organoids were prepared as previously described^26^. Immunohistochemical analysis of MAOA, Calbindin D28K, IL-6, and STAG2 expression in chronic prostate organoids and human prostate biopsy specimens was performed with the tissues, which were fixed with 10% formalin in Phosphate-Buffered Saline (PBS) at room temperature for at least 48 h, embedded in paraffin, and then cut into serial 4-µm-thick sections. Following deparaffinization in xylene and rehydration in a graded series of ethanol concentrations (from 100 to 70%), tissue sections were microwaved twice for 5 min at 95 °C in citrate buffer as a pretreatment, and then washed three times in PBS for 3 min at room temperature. Endogenous peroxidase activity was blocked by incubation with 3% hydrogen peroxide in methanol for 10 min at room temperature. The tissue sections were then washed three times with PBS and blocked with a protein-blocking solution [5% normal horse serum (cat. no. H1138; Sigma-Aldrich; Merck KGaA) and 1% normal goat serum (cat. no. G6767; Sigma-Aldrich; Merck KGaA) in PBS] at room temperature for 10 min. After washing three times with PBS, primary antibodies (MAOA dilution, 1:300; STAG2 dilution, 1:100; Calbindin D28K dilution, 1:50; and IL-6 dilution, 1:1000) were added to the slides, which were then incubated in humidified boxes at 4 °C overnight. The slides were washed thrice with PBS. After further incubation with the respective peroxidase-conjugated secondary antibodies (dilution, 1:500) at room temperature for 1 h, a positive reaction was detected by exposing the slides to stable 3,3′-diaminobenzidine at room temperature for 5–10 min. To visualize the nuclei, the slides were counterstained with hematoxylin (cat. no. 1.09249.0500; Merck KGaA), which was added as an undiluted solution at room temperature for 15 s. Immunohistochemical staining was performed according to standard protocols as previously described^27–29^, with minor modifications.

The immunohistochemical (IHC) staining was semi-quantitatively evaluated by two independent observers blinded to the clinical data. To ensure a clinically relevant comparison, samples were categorized into two groups for each marker based on staining intensity and distribution, following established scoring conventions (e.g., TCGA guidelines).

Specifically, MAOA expression was classified into ‘high positive’ and ‘medium positive’ groups. For CALBI1, IL-6, and STAG2, samples were dichotomized into ‘positive’ and ‘negative’ groups. Statistical analysis was performed using the Fisher’s exact test to assess the association between these IHC categories and clinical pain severity. A p-value of < 0.05 was considered statistically significant.

### MAOA activities

MAOA activity was measured using serum samples obtained from patients with CPPS who were distinct from those whose tissues were used for organoid generation or immunohistochemical analysis in the context of CP/CPPS.

MAOA activity was measured using an MAO assay kit (Cell Biolabs, San Diego, CA, USA) according to the manufacturer’s instructions^30^.

The MAOA activity in the samples was measured using a spectrophotometric assay kit with different substrates, and hydrogen peroxide was formed. MAOA reacts with its substrates to generate hydrogen peroxide (H2O2). In the presence of horseradish peroxidase (HRP), the Colorimetric Probe reacts with H2O2 to produce a red/pink product. Absorbance was measured at 540 nm using a microplate reader. The results were expressed in mU/mg protein.

### Statistical analysis

Comparisons between groups were performed using the chi-square test for categorical variables using JMP PRO18. Two-way repeated measures ANOVA (type III sum of squares) was conducted to assess the effects of treatment over time between the two groups using GraphPad Prism version 10 (GraphPad Software, LLC, Boston, MA, USA). All tests were two-sided, and *p* < 0.05 was considered statistically significant.

## Supplementary Information

Below is the link to the electronic supplementary material.

Supplementary Material 1

Supplementary Material 2

## Data Availability

The datasets generated and analyzed in the current study are available from the corresponding author upon reasonable request.
