# Supplementary material for: Patient-derived prostate organoids identify MAOA as a disease severity-associated molecular marker in chronic pelvic pain syndrome
Source: Sci Rep. 2026 May 19;16:22846. doi: 10.1038/s41598-026-53351-4 (PMC13389521; doi:10.1038/s41598-026-53351-4)
Supplement: Supplementary file 1 — Supplementary Material 1 [file 41598_2026_53351_MOESM1_ESM.pdf]

マイクロアレイデータ解析報告書

広島大学 自然科学研究支援開発センター

生命科学実験部門 生命科学機器分析部 NGS データ解析チーム

2019 年 9 月 10 日

バージョン v0.3-190910

目次

■依頼内容と結果について... 3

■Step 1. サンプル QC(TAC)..5

■Step 2. 定量化(GeneSpring)..5

■Step 3. 発現量の低い遺伝子を除去(GeneSpring)..5

■Step 4. クラスタリング(GeneSpring)..6

■Step 5. 統計解析(GeneSpring)..7

■Step 6. Fold Change(GeneSpring)...8

■Step 7. パスウェイ解析(IPA)..9

■Step 8. コントロール同士の比較(GeneSpring,IPA).......................................13

■依頼内容と結果について

●解析目的　マイクロアレイ GeneChip で測定したデータ 27 サンプルの発現量算出を行い、重度/中等度・軽度について変動遺伝子を検出する。

●使用データ（CEL 形式）

Sample ID	Condition	平均化後のサンプル名
No1_Co	重度	2_Co
No1_Evi	重度	2_Evi
No1_Tad	重度	2_Tad
No3_Co	中等度	1_Co
No3_Evi	中等度	1_Evi
No3_Tad	中等度	1_Tad
No4_Co	軽度	1_Co
No4_Evi	軽度	1_Evi
No4_Tad	軽度	1_Tad
No5_Co	重度	2_Co
No5_Evi	重度	2_Evi
No5_Tad	重度	2_Tad
No7_Co	中等度	1_Tad
No7_Evi	中等度	1_Tad
No7_Tad	中等度	1_Tad
No8_Co	軽度	1_Tad
No8_Evi	軽度	1_Tad
No8_Tad	軽度	1_Tad
No9_Co	軽度	1_Tad
No9_Evi	軽度	1_Tad
No9_Tad	軽度	1_Tad
No10_Co	軽度	1_Tad
No10_Evi	軽度	1_Tad
No10_Tad	軽度	1_Tad
No11_Co	重度	2_Tad
No11_Evi	重度	2_Tad
No11_Tad	重度	2_Tad

●解析に使用したソフトウェアとバージョン
1.Transcriptome Analysis Console(TAC)(Thermo Fisher Scientific)
Version:4.0.1.36
2.GeneSpring（Agilent Technologies）
Version:14.9.1 –GX -PA
3.IPA(Tomy Digital Biology)
Version:47547484

●解析結果 以下の 11 ファイルです。
・1.Expression List_2.xlsx
「Step 2. 定量化(GeneSpring)」後の結果で、全遺伝子の発現量ファイルです。

・1&2,3_ANOVA_軽度中等度_only Evi.xlsx
・1&2,3_ANOVA_軽度中等度_only Tad.xlsx
・1&2,3_ANOVA_重度_only Evi.xlsx
・1&2,3_ANOVA_重度_only Tad.xlsx
「Step 5. 統計解析(GeneSpring)」後の結果です。

・1&2,3_FC2_軽度中等度_only Evi.xlsx
・1&2,3_FC2_軽度中等度_only Tad.xlsx
・1&2,3_FC2_重度_only Evi.xlsx
・1&2,3_FC2_重度_only Tad.xlsx
「Step 6. Fold Change(GeneSpring)」後の結果です。

・1&2,3_FC2_control.xlsx
・1&2,3_IPA_FC2_control.pdf
「Step 8. コントロール同士の比較(GeneSpring,IPA)」後の結果です。

■Step 1. サンプル QC(TAC)

Hybridization Controls Threshold(ハイブリカクテルに添加したコントロール遺伝子の発現量パターン)、Pos vs Neg AUC Threshold(定義された 100 遺伝子の Exon 部分(Positive)と Intron 部分(Negative)のシグナル強度比 0.7 以上)の 2 項目についてはPASS しており問題ないデータが取得できていました。Labeling Controls Threshold(サンプル調整時に添加したコントロール遺伝子の発現量パターン)に QC 項目ついては No10_Co と No10_Evi で OUT しておりましたが、メーカーに確認したところ結果に影響ないとのことでしたので、解析に使用させて頂きます。

解析パラメーターは以下の通り。
Array Type: Clariom_S_Human
Analysis Type: Expression (Gene)
Analysis Version: version 2
Summarization Method: Gene Level - SST-RMA
Pos vs Neg AUC Threshold: 0.7
Genome Version: hg38 (Homo sapiens)
Annotation: Clariom_S_Human.r1.na36.hg38.a1.transcript.csv

■Step 2. 定量化(GeneSpring)

解析パラメーターは以下の通り。
Summarization Algorithm: ExonRMA
Technology: Affymetrix.TranscriptomeChip.Clariom_S_Human_na36_hg38_2016-06-21
Normalization: None
Baseline Transformation: median of all samples

■Step 3. 発現量の低い遺伝子を除去(GeneSpring)

全サンプルについて発現量が低い遺伝子を除去しました。
これにより遺伝子数は 21448 個→18703 個となりました。
Step 4 以降の解析は全てこのリストに対して行っています。

解析パラメーターは以下の通り。
Entity List : All Entities
Interpretation : 1&2,3
Experiment : all_RMA

Data to filter on : Raw Data

Lower cut-off: 20.0

Upper cut-off: 100.0

Entities where at least 1 out of 27 samples have values within cut-off

■Step 4. クラスタリング(GeneSpring)

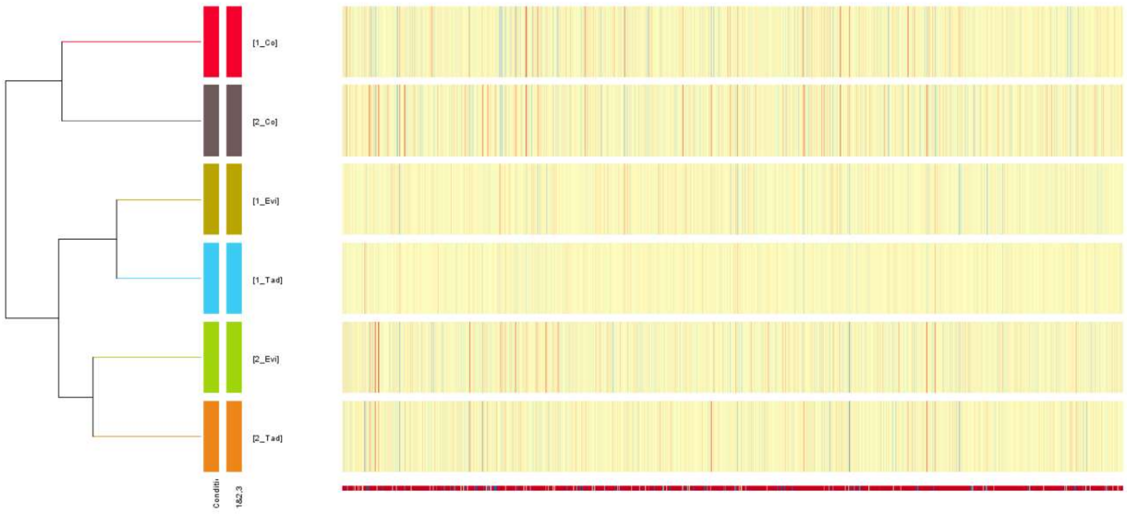

Fig.1. クラスタリング.

解析パラメーターは以下の通り

Created from Advanced Analysis operation: Clustering:

Entity List: Filtered on Expression (20.0 - 100.0)th Percentile in the Raw Data

Interpretation: 1&2,3

Experiment: all_RMA

Clustering Algorithm: Hierarchical

Clustered By: Normalized intensity values

Clustered On: Conditions

Similarity Measure: Euclidean

Linkage Rule: Wards

Cluster Within Conditions: No

■Step 5. 統計解析(GeneSpring)

軽度/中等度・重度それぞれについて平均化し、統計解析(ANOVA)を行いました。

まず平均化後の全 6 群について ANOVA 解析で p-value を算出し、p-value≦0.05 でカットオフした結果 468 個の遺伝子が検出されました(ANOVA は多条件比較する際に使用され 468 個は全組み合わせで検出された遺伝子の総数です)。

この結果、それぞれの組み合わせの遺伝子数は以下のようになりました。

Table1. ANOVA p-value≦0.05 の遺伝子数

	軽度/中等度	重度
[Evi] vs [Co]	362	149
[Tad] vs [Co]	318	139
共通	295	87

このうち Evi のみまたは Tad のみで有意差があった遺伝子のリストを、数が多いので別添えファイルにさせていただいています。以下のファイルです。

・1&2,3_ANOVA_軽度中等度_only Evi.xlsx
・1&2,3_ANOVA_軽度中等度_only Tad.xlsx
・1&2,3_ANOVA_重度_only Evi.xlsx
・1&2,3_ANOVA_重度_only Tad.xlsx

下に記載しているのは Evi,Tad それぞれ軽度/中等度・重度間で共通していた遺伝子で、黄色になっているものは炎症に関連する遺伝子です。Tad の方では STAG2 がやはり入ってきています。MAOA は今回の解析では軽度/中等度で入っていませんでした。

Table2-1. ANOVA 解析で Evi のみで有意差があったもののうち軽度/中度・重度間で共通する遺伝子

genesymbol	entrezgene	Type
AGPS	8540	alkylglyceronephosphatesynthase
ITGB6	3694	integrinbeta6
ARSB	411	arylsulfataseB
KIF3A	11127	kinesinfamilymember3A
TRIM27	5987	tripartitemotifcontaining27
AVL9	23080	
SEMA3C	10512	semadomain,immunoglobulindomain(Ig),shortbasicdomain,sec

		reted,(semaphorin)3C
ACP2	53	acidphosphatase2,lysosomal
RCCD1	91433	RCC1domaincontaining1
KIF3B	9371	kinesinfamilymember3B

Table2-2. ANOVA 解析で Tad のみで有意差があったもののうち軽度/中度・重度間で共通する遺伝子

genesymbol	entrezgene	Type
STAG2	10735	stromalantigen2
COMMD9	29099	COMMdomaincontaining9
VPS4A	27183	vacuolarproteinsorting4homologA(S.cerevisiae)
MARCH2	51257	membraneassociatedringfinger2

解析パラメーターは以下の通り。

Created from Advanced Analysis operation: significance Analysis.

Experiment: all_RMA

corrected p-value cut-off:0.05

Post-hoc test: tukeyHSD

Selected Test : Oneway ANOVA

Entity List: Filtered on Expression (20.0 - 100.0)th Percentile in the Raw Data

Interpretation: 1&2,3

p-value computation: Asymptotic

Multiple Testing Correction: Benjamini-Hochberg

■Step 6. FoldChane(GeneSpring)

ANOVA 解析と同様に平均化した後、[Evi] vs [Co], [Tad] vs [Co]の 2 パターンで、2 倍以上発現量差があった遺伝子を算出しました(全部で 4 パターン)。

その後 軽度/中等度・重度それぞれで Evi のみまたは Tad のみで変動していた遺伝子を抽出しました。以下のファイルです。

・1&2,3_FC2_軽度中等度_only Evi.xlsx (113 個)

・1&2,3_FC2_軽度中等度_only Tad.xlsx (24 個)

・1&2,3_FC2_重度_only Evi.xlsx (112 個)

・1&2,3_FC2_重度_only Tad.xlsx (105 個)

解析パラメーターは以下の通り。

Fold change >= 2.0

Entity List : Filtered on Expression (20.0 - 100.0)th Percentile in the Raw Data

Interpretation : 1&2,3

Experiment : all_RMA

Fold-Change cut-off : 2.0

Pairing option : Pairs of conditions

Condition pairs :

[1_Evi] vs [1_Co]

[1_Tad] vs [1_Co]

[2_Evi] vs [2_Co]

[2_Tad] vs [2_Co]

Minimum number of pairs:1 out of 4condition pairs.

■Step 7. パスウェイ解析(IPA)

Step 6.で抽出した 4 つの遺伝子リストを IPA にエクスポートし、パスウェイ解析を行いました。また炎症と関連の有る遺伝子は以下の通りです。

このうち Evi のみで発現量差があったもののうち軽度/中等度・重度間で共通する遺伝子は黄色、Tad でのみ発現量差があったもののうち軽度/中等度・重度間で共通する遺伝子が緑色です。重度のみで差があった遺伝子の内いくつかピックアップして調べるのも有効かと思います。

Table3-1. IPA_FC2_軽度/中等度_only Evi_inflammation

Molecule Name	Entrez Gene ID for Human
ADM	133
AGR2	10551
AQP9	366
BCL6	604
C3	718
CD36	948
CPE	1363
CXCL1	2919
DDX58	23586
DSC1	1823

ERBB3	2065
FGFR3	2261
GSDMC	56169
IFNAR2	3455
IGFBP3	3486
IL32	9235
IL37	27178
ITGAV	3685
KLK13	26085
LCE3D	84648
LIPA	3988
MMP2	4313
NUPR1	26471
PGLYRP3	114771
PRSS3	5646
RARG	5916
RASSF5	83593
RNF14	9604
RNF39	80352
SGCB	6443
SPOCK1	6695
STEAP4	79689
TGM3	7053
TNC	3371
TPM1	7168
UGT1A1	54658
VNN1	8876
ZBTB16	7704

Table3-2. IPA_FC2_軽度/中等度_only Tad_inflammation

Molecule Name	Entrez Gene ID for Human
CYP4F11	57834
DEFB4A/DEFB4B	1673|100289462
DKK1	22943
IL36A	27179

INPP5D	3635
MAOA	4128
ROS1	6098
SERPINB3	6317

Table3-3. IPA_FC2_重度_only Evi_inflammation

Molecule Name	Entrez Gene ID for Human
ABCC4	10257
ADM	133
ADRB2	154
AGT	183
ATF3	467
BDKRB2	624
BMP4	652
C3	718
CCN1	3491
CCN2	1490
CEACAM7	1087
COL3A1	1281
CPE	1363
CXCL16	58191
CYP4F11	57834
DUSP10	11221
FN1	2335
FRZB	2487
FSTL1	11167
IDO1	3620
IFIH1	64135
IFNAR2	3455
INPP5D	3635
ITGB6	3694
mir-198	406975
MMP7	4316
MMP13	4322

MXI1	4601
NMI	9111
PDE3B	5140
PDGFRA	5156
PFKFB3	5209
PIM1	5292
PLAU	5328
PMP22	5376
RNF39	80352
SAA1	6288
SLC1A1	6505
SLC26A4	5172
SLC44A4	80736
SNHG29	125144
VEGFA	7422

Table3-4. IPA_FC2_重度_only Tad_inflammation

Molecule Name	Entrez Gene ID for Human
ANGPTL4	51129
AZGP1	563
CD36	948
CDH13	1012
CFB	629
CFH	3075
CKMT1A/CKMT1B	1159|548596
CTSS	1520
CTSV	1515
DPP4	1803
FABP5	2171
FDPS	2224
HSD11B1	3290
IL6	3569
IL1RN	3557
ITGA5	3678
KLK7	5650

KLK13	26085
KRT1	3848
LGALS9B	284194
LY75	4065
LY6G6C	80740
MUCL1	118430
PGLYRP3	114771
PLA2R1	22925
PTGS2	5743
RRM2	6241
SERPINA3	12
SERPINB2	5055
SERPINB3	6317
SERPINB4	6318
SERPINB13	5275
TGM3	7053
TOP2A	7153
UGT1A1	54658
ANGPTL4	51129
AZGP1	563
CD36	948

■Step 8. コントロール同士の比較(GeneSpring)

重度のコントロール vs 軽度/中等度のコントロールで FoldChange2 解析を行い、変動があった遺伝子を抽出しました。以下のファイルです。

・1&2,3_FC2_control.xlsx

またこのリストについてパスウェイ解析も行いました。以下のファイルです。

・1&2,3_IPA_FC2_control.pdf

そのリストと Evi のみで変動があったものと Tad のみで変動があったものを比べ、重複する遺伝子を以下の表に記載しています。黄色は炎症関連遺伝子です。

Table4-1. FC2_Co and FC2_軽度/中度_only Evi

Molecule Name	Entrez Gene ID for Human
LCE6A	448835
CXCL1	2919
STC2	8614
FRMD3	257019
MMP2	4313

Table4-2. FC2_Co and FC2_軽度/中度_only Tad

Molecule Name	Entrez Gene ID for Human
COL6A3	1293
ROS1	6098
DEFB4B	1E+08
HAS2	3037

Table4-3. FC2_Co and FC2_重度_only Evi

Molecule Name	Entrez Gene ID for Human
CAMK1G	57172
AGT	183
COL3A1|MIR3606	1281///100500837
FN1	2335
COL6A3	1293
PCDH18	54510
PDGFRA	5156
FAM105A	54491
HIST1H2AG	8969
IDO1	3620
COLEC10	10584
NRK	203447
PDE3B	5140
IFITM1	8519
ABCC4	10257
BMP4	652
BDKRB2	624
FBN1	2200

SALL1	6299

Table4-4. FC2_Co and FC2_重度_only Tad

Molecule Name	Entrez Gene ID for Human
CFH	3075
HSD11B1	3290
CTSS	1520
LY75-CD302	100526664///9936///4065
DPP4	1803
RFTN2	130132
RASGRF2	5924
CFB	629
IL6	3569
GNG11	2791
GLIPR1	11010
C1S	716
NID2	22795
SLC14A1	6563
