# Supplementary material for: Patient-derived prostate organoids identify MAOA as a disease severity-associated molecular marker in chronic pelvic pain syndrome
Source: Sci Rep. 2026 May 19;16:22846. doi: 10.1038/s41598-026-53351-4 (PMC13389521; doi:10.1038/s41598-026-53351-4)
Supplement: Supplementary file 2 — Supplementary Material 2 [file 41598_2026_53351_MOESM2_ESM.docx]

**Supplementary Figure S2**.

IL-6 expression increased in mild and moderate cases but decreased in severe cases (**D**). STAG2 tended to decrease following both treatments (**E**).

**Supplementary Figure S3**.

IL-6 (**D**) and STAG2 (**E**) showed no significant association with pain severity.

**Supplementary Table 3.　Immunohistochemical expression of four candidate genes by symptom severity in CPPS.**

Comparison of positive and negative immunohistochemical staining for MAOA, CALB1, IL-6, and STAG2 between patients with mild symptoms (Score ≤1) and those with moderate to severe symptoms (Score ≥2). MAOA and CALB1 showed significant associations with higher symptom severity, whereas IL-6 showed a non-significant trend and STAG2 showed no difference.


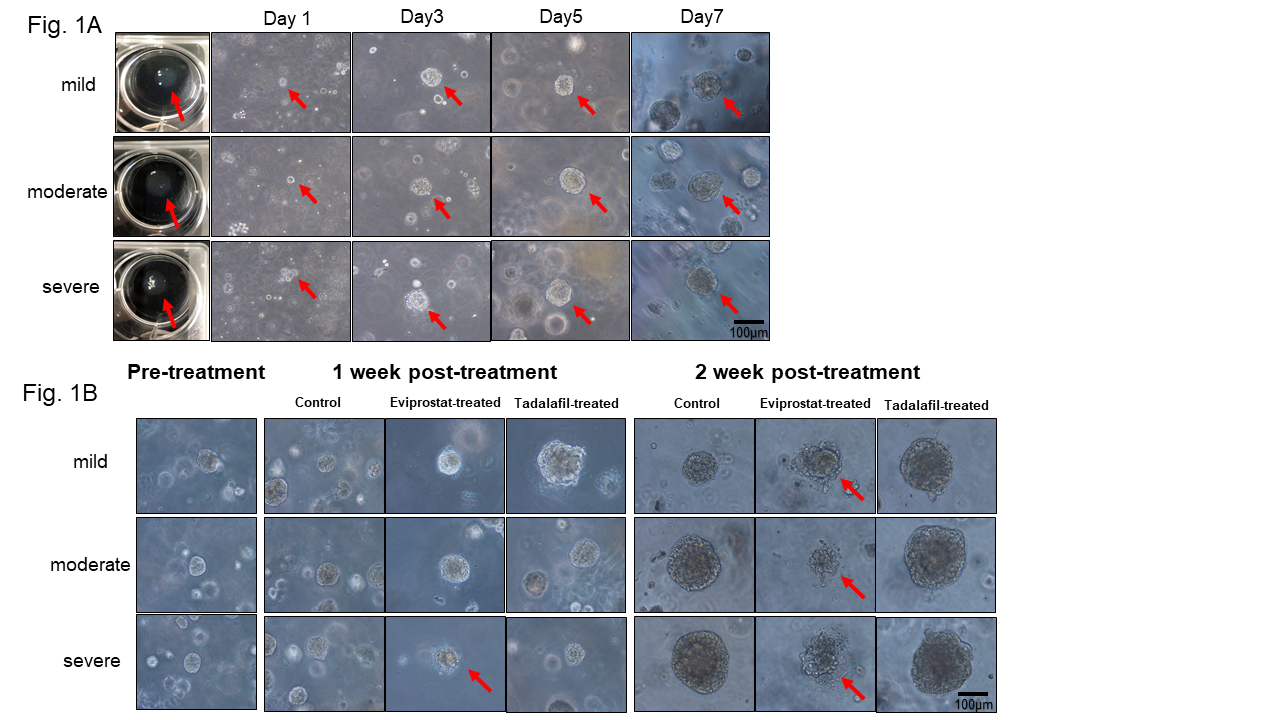


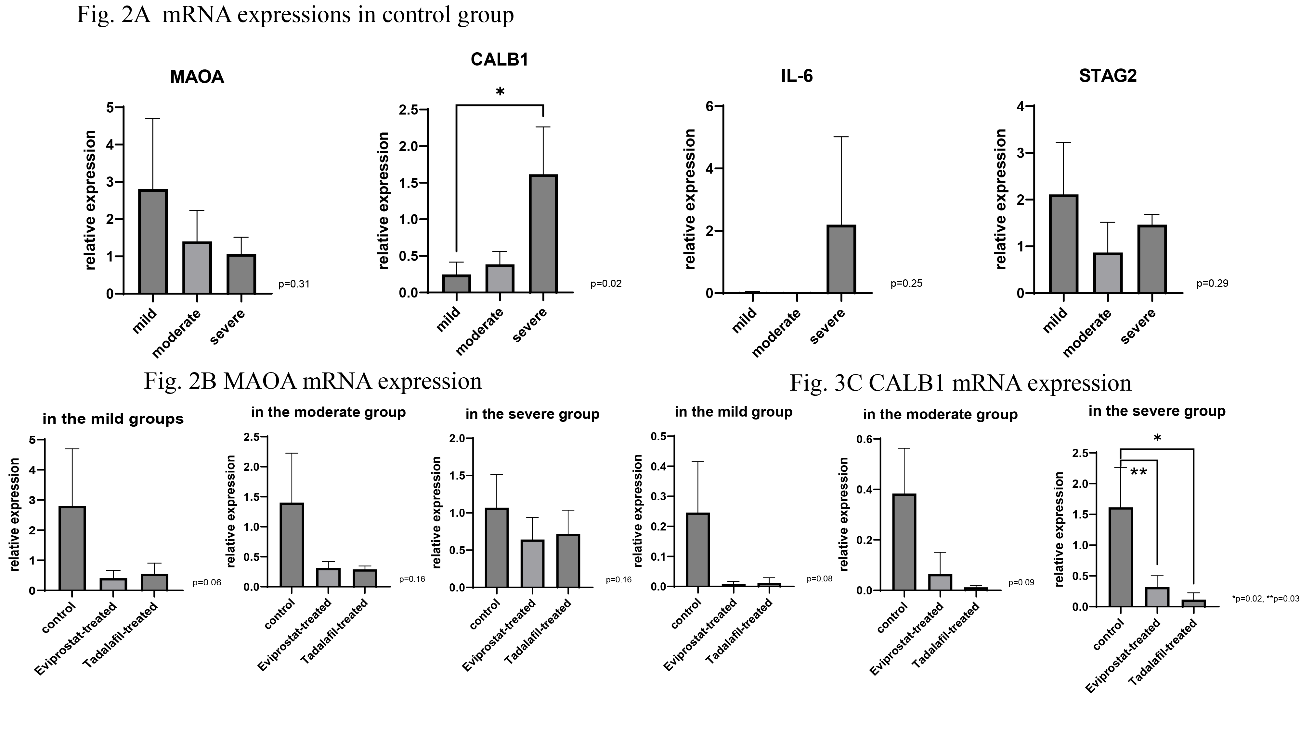


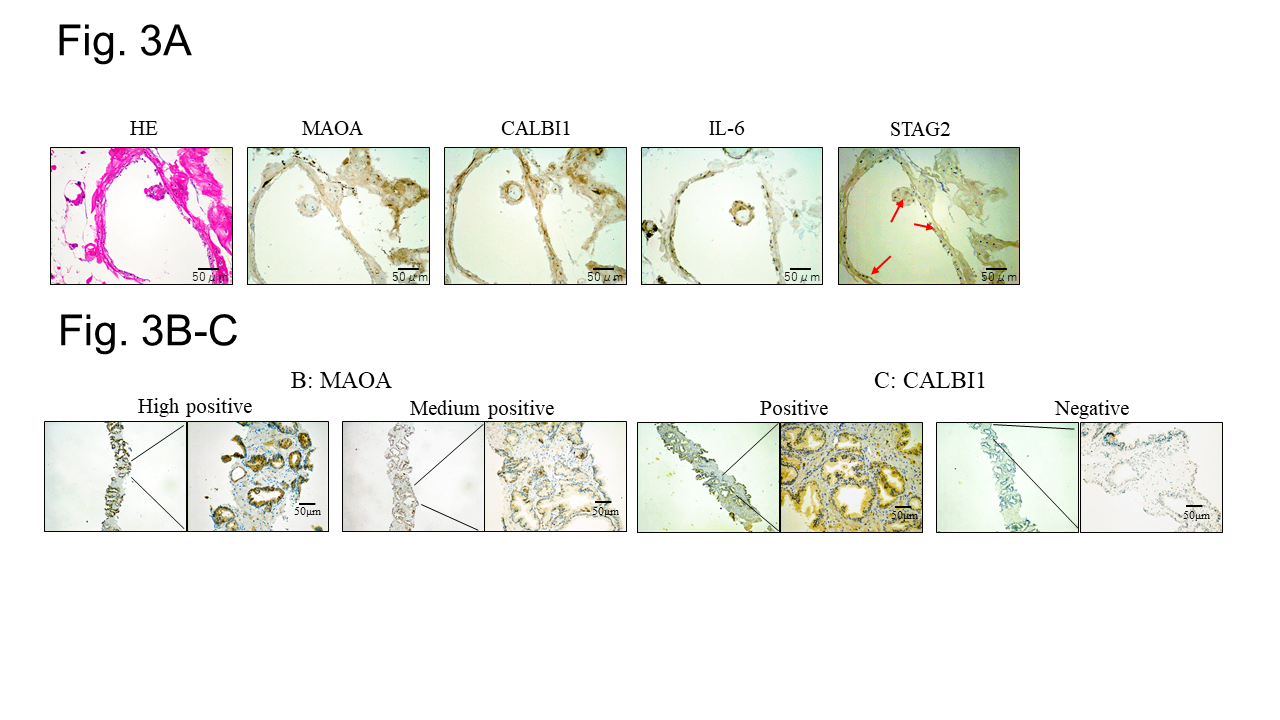


Supplementary Figure1


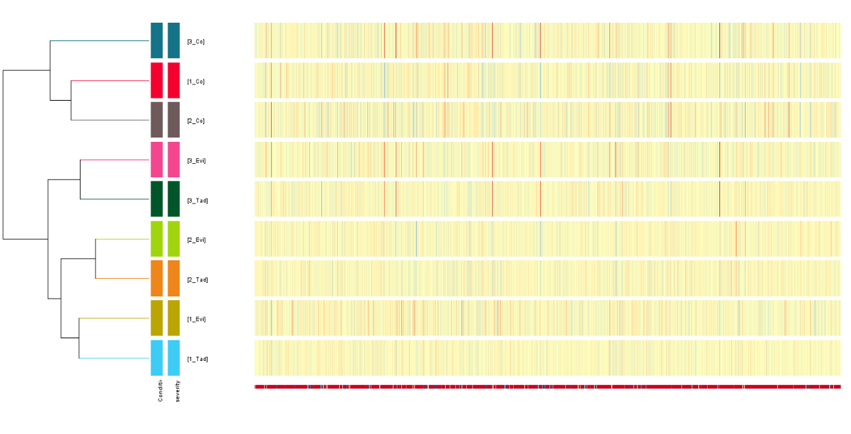


Supplementary Figure2


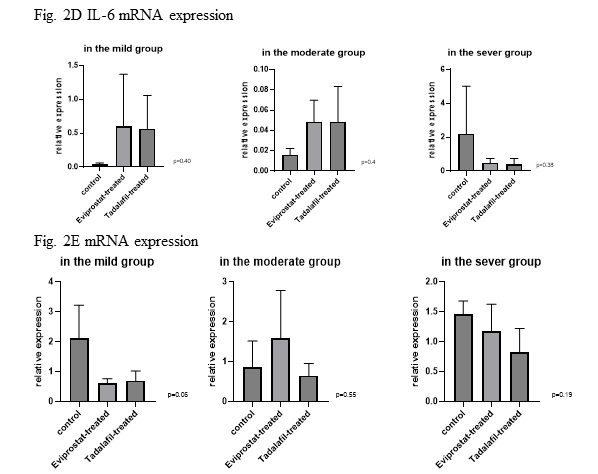


Supplementary Fgure3


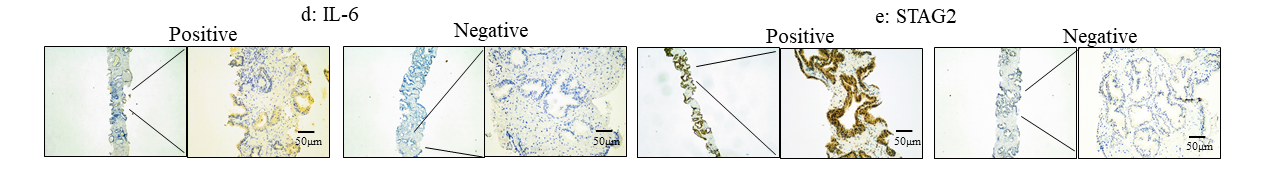


Supplementary Table2

Supplementary Table3
